# Quantitative Morphological Analysis of Filamentous Microorganisms in Cocultures and Monocultures: *Aspergillus terreus* and *Streptomyces rimosus* Warfare in Bioreactors

**DOI:** 10.3390/biom11111740

**Published:** 2021-11-22

**Authors:** Anna Ścigaczewska, Tomasz Boruta, Marcin Bizukojć

**Affiliations:** Department of Bioprocess Engineering, Faculty of Process and Environmental Engineering, Lodz University of Technology, ul. Wolczanska 213, 90-924 Lodz, Poland; tomasz.boruta@p.lodz.pl (T.B.); marcin.bizukojc@p.lodz.pl (M.B.)

**Keywords:** cocultures, filamentous microorganisms, morphology, image analysis

## Abstract

The aim of this study was to quantitatively characterize the morphology of the filamentous microorganisms *Aspergillus terreus* ATCC 20542 and *Streptomyces rimosus* ATCC 10970, cocultivated in stirred tank bioreactors, and to characterize their mutual influence with the use of quantitative image analysis. Three distinct coculture initiation strategies were applied: preculture versus preculture, spores versus spores and preculture versus preculture with time delay for one of the species. Bioreactor cocultures were accompanied by parallel monoculture controls. The results recorded for the mono- and cocultures were compared in order to investigate the effect of cocultivation on the morphological evolution of *A. terreus* and *S. rimosus*. Morphology-related observations were also confronted with the analysis of secondary metabolism. The morphology of the two studied filamentous species strictly depended on the applied coculture initiation strategy. In the cocultures initiated by the simultaneous inoculation, *S. rimosus* gained domination or advance over *A. terreus*. The latter microorganism dominated only in these experiments in which *S. rimosus* was introduced with a delay.

## 1. Introduction

The application of microbial cocultures, understood as the simultaneous cultivation of two or more microorganisms in the individual closed environment, is a well-known strategy to improve their metabolic diversity. In natural habitats, filamentous microorganisms grow in various multispecies communities and establish interrelationships which can be based on the competition or coexistence [1,2]. In the first case, antifungal and antibacterial substances are produced by filamentous microorganisms in order to increase their advantage over other hostile microorganisms. In the second case, the compounds produced by one of the species can be biotransformed by the symbiotic microorganism. Owing to the aforementioned multilevel dependencies, microorganisms cultivated in the artificial laboratory bioreactor cocultures may activate biosynthetic pathways that are hidden or cryptic in the monocultures [3,4,5,6,7,8,9]. Therefore, the application of the cocultures can result in the enhancement of metabolites production [10].

Some of the better known cocultures are those of genera *Aspergillus* and *Streptomyces* due to their broad secondary metabolic repertoires. In recent years, numerous studies in this field were conducted. However, the cocultures of *Aspergillus terreus* and *Streptomyces rimosus*, chosen to be investigated in the present work, have not been investigated so far [8,11,12]. The results of the recent *Aspergillus* and *Streptomyces* coculture studies are presented in Table 1. What is more, only three results relate directly to microbial morphology and none of the listed reports dealt with the experiments made in stirred tank bioreactors. The broadest morphological description of the cocultured microorganisms was made by Siemieniewicz and Schrempf [13]. They conducted static flask cocultivations (without shaking) of *Aspergillus proliferans* and *Streptomyces olivaceoviridis* to prevent pellets formation. The mixture of *A. proliferans* and *S. olivaceoviridis* spores in the coculture provoked the germination and growth of the cultivated microorganisms in the medium without any carbon source. In the monocultures run in the same medium, either the spores of *A. proliferans* or *S. olivaceoviridis* did not enter the germination phase. Growth of the fungus was initiated by chitinase ChiO1 produced by *S. olivaceoviridis*. Morphological observations of germination were made with the phase-contrast optical microscope. In a different study, Caceres et al. [14] investigated the bioagents that reduce the concentration of carcinogenic aflatoxin B_1_. This mycotoxin is produced by *Aspergillus flavus*, a food-contaminating fungus. Agar plates with *A. flavus* and *Streptomyces roseolus* cocultures demonstrated the *S. roseolus-*dependent inhibition of *A. flavus* gene cluster responsible for aflatoxin B_1_ biosynthesis. Fungal morphology was described on the basis of Scanning Electronic Microscopy (SEM) observations. The main change caused by the introduction of *S. roseolus* into *A. flavus* culture was the increase in fungal spores quantity. In the research conducted by Yu et al. [15] microbial morphology was also investigated with SEM, however they presented images originating only from the cocultures, without referring to the monoculture controls, and there was no demonstration of quantitative morphological data. The results showed that the physical contact of *Aspergillus flavipes* and *Streptomyces* sp. in the cocultures was required for inducing the production of the bioactive cytochalasans (Table 1). A similar approach was presented by Schroeckh et al. [3]. In their research, the direct contact between the cultivated microorganisms, *Aspergillus nidulans* and *Streptomyces hygroscopicus*, was one of the ways for the elicitation of a silent polyketide synthase (PKS) gene cluster. Nevertheless, this paper focused on the physical interactions between the cocultured microorganisms instead of their morphology. König et al. [16], as one of very few research teams, showed the comparison of monocultures with cocultures. They conducted *Aspergillus fumigatus* and *Streptomyces rapamycinicus* agar plate cocultivations. Physical interactions between these two microorganisms led to the biosynthesis of fumicycline A, a prenylated polyphenol encoded by the PKS gene cluster that remained silent under monoculture conditions. The differences between *A. fumigatus* monoculture and *A. fumigatus* and *S. rapamycinicus* coculture were shown with the use of scanning electron micrographs. It should be mentioned, however, that the SEM technique was not the only method of coculture observation. Optical and fluorescence microscopy were used by Wu et al. [17] and Stroe et al. [18] to demonstrate the intimate interactions between cocultured microorganisms. However, their research was not specifically targeted at morphological issues. Upon literature review one can find that no quantitative studies linking the bioproduction and morphology in cocultures have been conducted so far. Nevertheless, it is well known that in the bioprocesses involving filamentous microorganisms’ morphology is one of the key factors influencing the production of metabolites and cultivation conditions, such as medium rheological properties or oxygen availability [19]. Therefore, deeper morphological studies of filamentous cocultures are crucial for the future development of bioproducts synthesis in these systems.

Numerous works on microbial cocultivation do not fill the lack of information about morphological development in submerged cocultures [13,20,21,22,23,24,25,26,27,28]. Only Wurster et al. [2] made an attempt to quantitatively describe the morphology of cocultured microorganisms in their research on *A. fumigatus* growth blocked by *Pseudomonas aeruginosa*. They proved that pyoverdine and pyocyanin produced by *P. aeruginosa* inhibited *A. fumigatus* branching and growth. Furthermore, bioreactor cocultures were almost uncharted due to the difficulties in controlling multispecies processes. Treloar et al. [29] worked on solving this problem, and as a result, they developed an approach of reinforcement learning. The method was based on artificial intelligence and could be used for maintaining cultivated microorganisms at the targeted levels. The issue of the metabolism of filamentous microorganisms in the stirred tank bioreactor cocultures was for the first time reported by Boruta et al. [30]. They studied the production of secondary metabolites and bioprocess kinetics in *A. terreus* and *S. rimosus* bioreactor cocultures and respective parallel monocultures of these microorganisms. Moreover, upon the analysis of the metabolic repertoire, the metabolically dominant microorganism was indicated in each conducted coculture. The present morphological studies deal with the same *A. terreus* and *S. rimosus* bioreactor cocultures and for this reason the comparison of the metabolism and morphological development of the cocultivated *A. terreus* and *S. rimosus* in stirred tank bioreactors became possible for the first time. All in all, the presented literature review shows how much research is still required to develop the cocultures for industrial processing and emphasizes the importance of the morphological studies focused on filamentous microorganisms.

The aim of the present study was to perform the first quantitative morphological analysis of the filamentous microorganisms *A. terreus* and *S. rimosus* cultivated in the stirred tank bioreactor cocultures and to correlate the obtained results with their respective metabolic profiles.

## 2. Materials and Methods

### 2.1. Strains

A fungus, *Aspergillus terreus* ATCC 20542, and an actinomycete, *Streptomyces rimosus* ATCC 10970, from the American Type Culture Collection (ATCC) (Lomianki, Poland) were cultivated. The microorganisms were stored on agar slants of the composition in accordance with the ATCC recommendations. Slants for *A. terreus* contained malt extract 20 g·L^−1^, casein peptone 5 g·L ^−1^ and agar 20 g·L^−1^. *S. rimosus* was maintained on the commercial medium Difco ISP Medium 2. The slants were made as follows: Sterile water with suspended spores of the selected microorganism was poured into sterile slants. Next, slants were thermostated at 26 °C for 10 days and were stored at 4 °C for no longer than 2 weeks.

### 2.2. Bioreactor Experiments

All cultivations were conducted in fully controlled stirred tank bioreactors of 5.5 L working volume (Sartorius B-Plus, Sartorius Stedim, Germany) at 30 °C, equipped with pH, redox potential and dissolved oxygen sensors. The level of dissolved oxygen in the cultivation medium was set at 20% and controlled by air flow rate and stirring speed. Their values were in the following ranges: flow rate from 1.5 l_air_ min^−1^ to 5.5 l_air_ min^−1^ and stirring speed from 220 min^−1^ to 300 min^−1^.

Each individual experiment denoted from ATSR2 to ATSR9 contained three simultaneous bioreactor cultivations:

(1) coculture of *A. terreus* and *S. rimosus*,
(2) monoculture of *A. terreus*,
(3) monoculture of *S. rimosus*.

The monocultures were the sources of the reference data for the cocultures.

### 2.3. Coculture Initiation Strategies and Cultivation Medium Compositions

The first stage of the study included 5 experiments (from ATSR2 to ATSR6) performed with the use of the following coculture initiation strategy: 24 h shaking flask precultures of *A. terreus* and *S. rimosus* were introduced to the bioreactor in the same moment. The precultures’ preparation was previously described by Boruta et al. [30]. Due to fact that the initial number of spores in the precultures should be equal to 10^9^ per liter, spores were counted in a Thoma cell counting chamber and their number corrected, if required.

In the second stage of the study, two experiments (ATSR7 and ATSR8) were conducted. The coculture inoculation strategy in these two experiments was altered. *A. terreus* preculture was introduced to the bioreactor at the beginning of the cultivation while *S. rimosus* preculture introduction was delayed by 24 h. Therefore, *S. rimosus* monoculture also began 24 h later in comparison to *A. terreus* monoculture.

In the last stage of the study, a single experiment (ATSR9) was performed. In this case, the bioreactors intended for the coculture and monocultures were simultaneously inoculated by *A. terreus* spores and *S. rimosus* spores (10^9^ spores per liter of the inoculum without prior preculture). As a result, the spores germinated in the bioreactor.

### 2.4. Media Compositions

Various cultivation medium compositions were used in the experiments. For the sake of eligibility, they are collected in Table 2. However, it should be emphasized that each of the three cultivations included in one experiment (coculture of *A. terreus* and *S. rimosus*, monoculture of *A. terreus*, monoculture of *S. rimosus*) were conducted in the same medium. Finally, if the experiments were started with precultures, their medium compositions were identical to those in the corresponding bioreactors.

### 2.5. Morphological Analysis

Morphological analysis was conducted on the cultivation broth, collected every 24 h. The samples were taken from the coculture and both monocultures in parallel within an individual experiment. Next, vital slides were prepared and subjected to microscopic observations with the use of light phase contrast microscope (OLYMPUS BX53, Olympus Corporation, Japan). The images of the observed microorganisms were taken by the high-resolution RGB digital camera (OLYMPUS DP27) and analyzed with the use of image analysis software (OLYMPUS cellSens Dimension Desktop 1.16, Olympus Corporation, Japan). As a result, three morphological parameters, namely the projected area (A), elongation (E) and morphology number (Mo) defined by Wucherpfennig et al. [37], were obtained. Their detailed description was provided by Kowalska et al. [38]. Mean values of morphological parameters were calculated on the basis of at least 30 mycelial or actinomycete objects to assure the statistically significant data. A t-Student distribution with significance level α = 0.05 was used to calculate the confidence bands and a statistical *t*-test was applied to confirm the differences between the selected morphological parameters.

#### Semiautomatic Image Processing

The semiautomatic image processing procedure began with the application the median filter and Sobel filter, to, respectively, smooth the edges of the objects and detect them. The next step was image segmentation, previously described in detail by Kowalska et al. [39]. The calculation of morphological parameters was conducted for *A. terreus* and *S. rimosus* separately. Even in the coculture, the objects were recognized and attributed to the studied species. This was performed using shape and size filters. Nevertheless, some of the *A. terreus* and *S. rimosus* objects turned out to be too similar for the automatic discrimination. In such cases, the operator made a decision regarding the species identification based on the observations of its morphological features. The last step of the image processing was the separation of two fractions within the data corresponding to a single microorganism: (1) the fully evolved pellets and (2) the hyphae and clumps. The shape and size filters were used for this purpose. The filter limit values were set by the operator depending on the morphology of the analyzed objects.

## 3. Results

The conducted experiments aimed at the identification of microbial morphology in coculture and finding the mutual influence between *A. terreus* and *S. rimosus*. Therefore, coculture initiation strategies and various cultivation medium compositions were taken into account. The full quantitative morphological analysis was performed by the calculation of the morphological parameters and discussed along with the changing culture conditions. Additionally, the morphological data were analyzed in the context of secondary metabolites production. Considering the cocultures, the terms “morphological domination” and “morphological advantage” were formulated to describe the “morphologically winning” microorganism. In the conducted cocultures, the morphological domination of one species was manifested by inhibiting the development of pellets of the competing microorganism. In the experiments in which the total destruction of pellets of one of the microorganisms did not occur, yet their sizes were significantly smaller, the term “morphological advantage” was applied.

### 3.1. Bioreactor Cocultures Initiated by Simultaneous Introduction of A. terreus and S. rimosus Precultures

**Figure 1:**
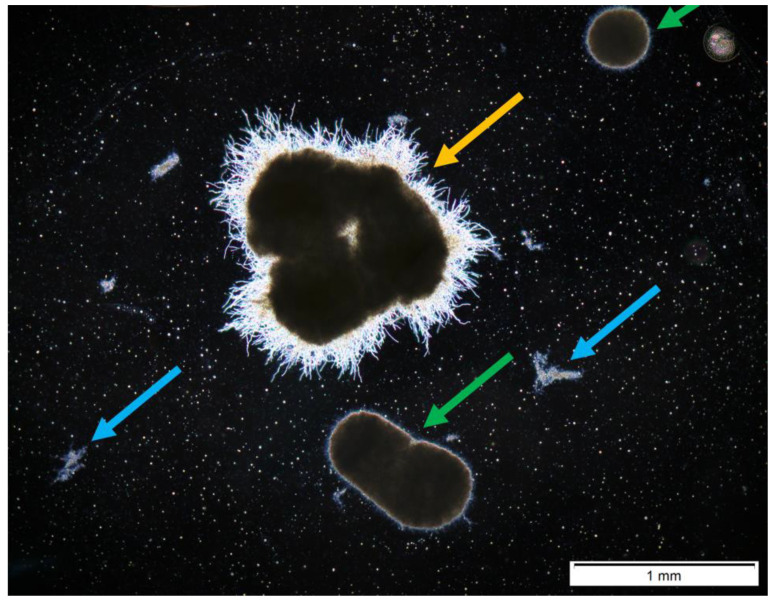
Exemplary microscopic image of the morphological objects snapped in 24 h of ATSR2 coculture: *A. terreus* fully evolved pellets (yellow arrow), *S. rimosus* fully evolved pellets (green arrows), and hyphae or clumps (blue arrows).

**Figure 2:**
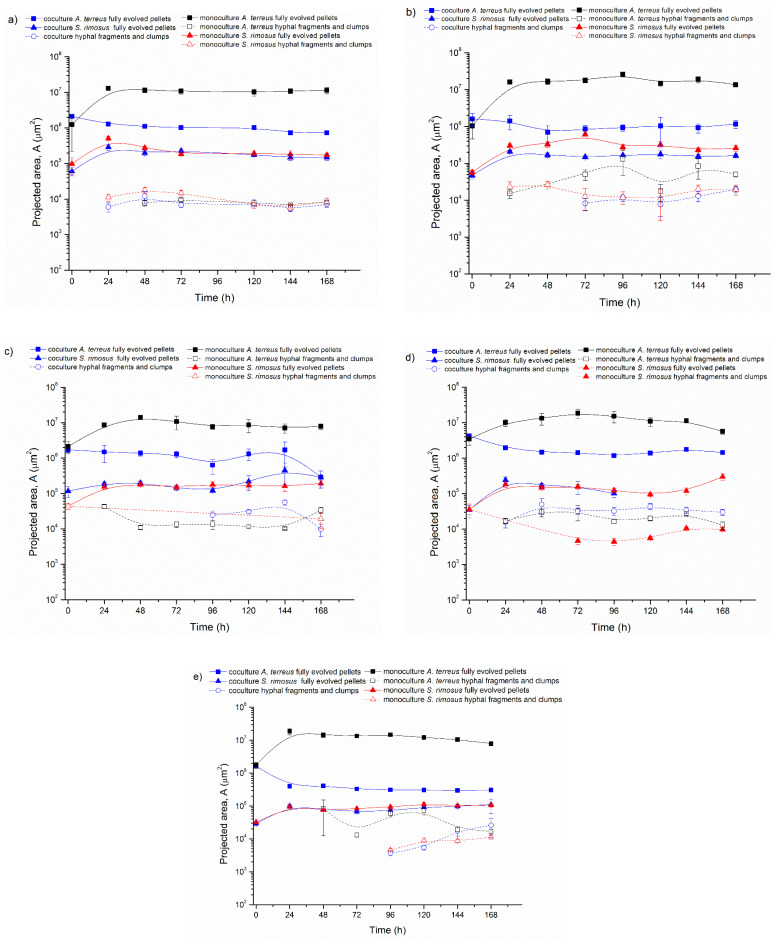
Temporal Changes of Projected Area (A) in: (**a**) ATSR2, (**b**) ATSR3, (**c**) ATSR4, (**d**) ATSR5, (**e**) ATSR6 Runs.

**Figure 3:**
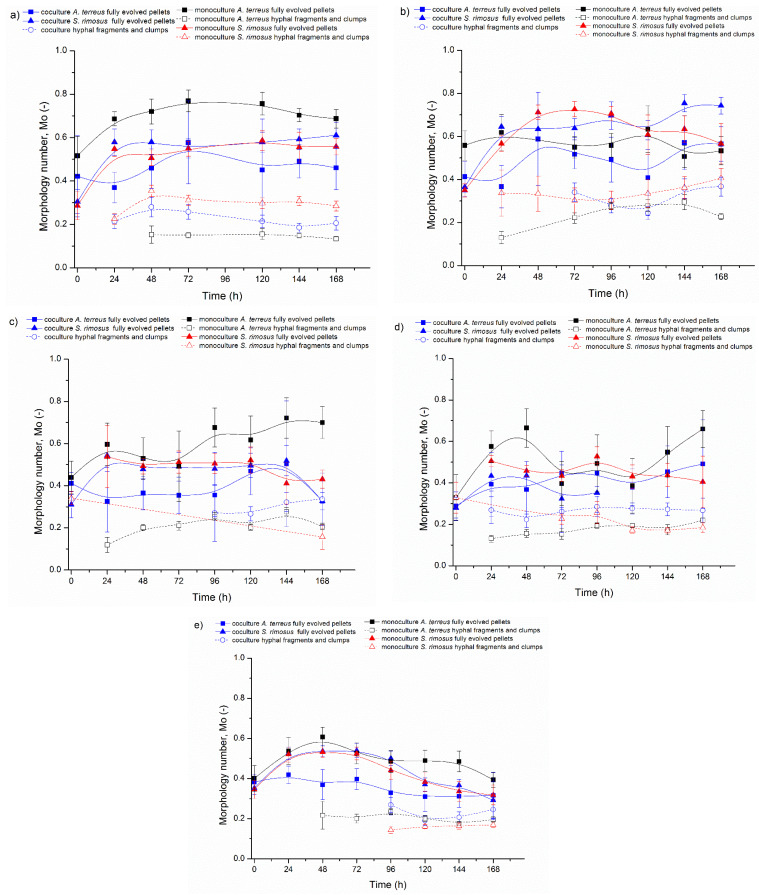
Temporal Changes of Morphology Number (Mo) in: (**a**) ATSR2, (**b**) ATSR3, (**c**) ATSR4, (**d**) ATSR5, (**e**) ATSR6 runs.

**Figure 4:**
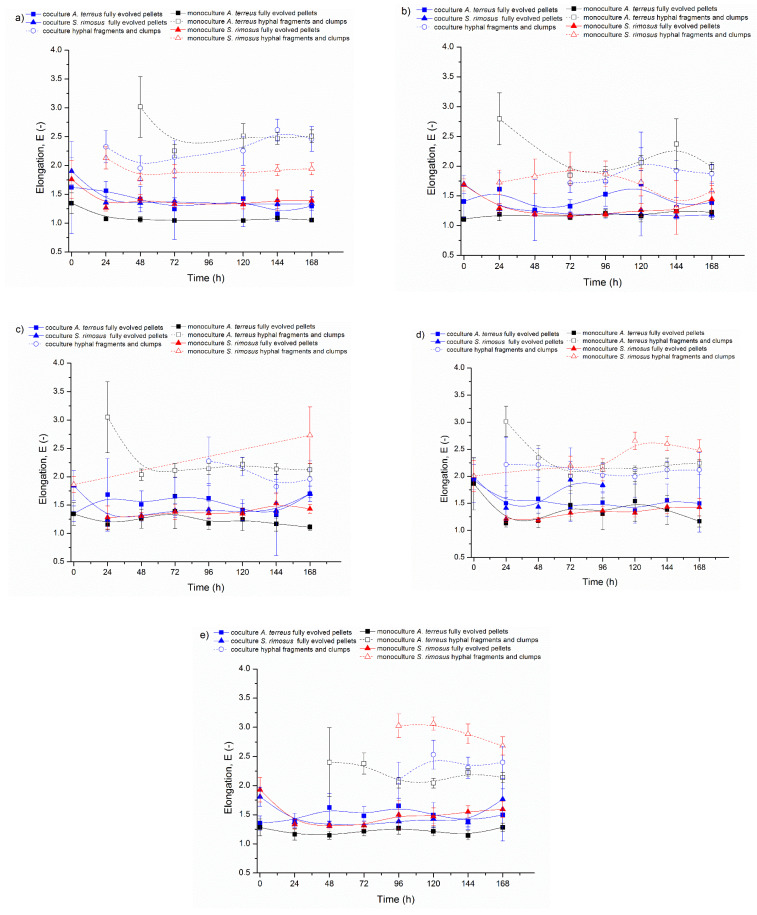
Temporal Changes of Elongation (E) in: (**a**) ATSR2, (**b**) ATSR3, (**c**) ATSR4, (**d**) ATSR5, (**e**) ATSR6 Runs.

Five experiments on ATSR2 to ATSR6 were conducted using *A. terreus* and *S. rimosus* 24 h precultures for the coculture initiation in the bioreactor. Each individual experiment contained three parallel bioreactor cultivations: one *A. terreus* and *S. rimosus* coculture and two monoculture controls of the tested microorganisms. The obtained results are presented in Figure 1, Figure 2, Figure 3, Figure 4. Figure 2 shows the temporal changes of projected area (A). Each individual graph in Figure 2 corresponds to one experiment and contains the calculated values from the coculture and both corresponding monocultures. In this graph, the projected area values were separated into the distinct fractions of mycelial objects: fully evolved pellets and hyphae or clumps. For the sake of clarity, the examples of objects representing these fractions are depicted in Figure 1. So, a fully evolved pellet of *A. terreus* is denoted with a yellow arrow, the fully evolved pellets of *S. rimosus* are marked with green arrows, and the hyphae or clumps undistinguishable with regard to the species are indicated with blue arrows.

The other two morphological parameters, morphology number (Figure 3) and elongation (Figure 4), are presented in these figures using the same scheme as above.

Both *A. terreus* and *S. rimosus* are filamentous species which form pellets in the submerged bioreactor cultivations. Nevertheless, the mechanisms of the formation of their pellets are different. *A. terreus* is an agglomerative species, while *S. rimosus* exhibits nonagglomerative pellet formation. However, during growth of these microorganisms, one can also observe hyphae and clumps which can be formed in two cases. First, it happens at the beginning of the cultivation before pellets become fully evolved. Second, hyphae and clumps can be formed when pellets are “shaved” due to mechanical forces present in the bioreactor or due to mycelium aging.

At the beginning of ATSR2 (Figure 2a) in the monoculture, the projected area of the fully evolved *A. terreus* pellets (marked with solid black squares) was equal to 1.2 × 10^6^ µm^2^ and increased over the next 24 h up to the value of 1.3 × 10^7^ µm^2^. In the next hours of the cultivation, this parameter slightly decreased (A = 1.1 × 10^7^ µm^2^ in 48 h) and then remained at this level. In the coculture, the projected area of *A. terreus* fully evolved pellets (marked with solid blue squares) showed a downward trend. Its initial value (at t = 0 h) was equal to 2.1 × 10^6^ µm^2^ and in the last 168 h it decreased to 7.3 × 10^5^ µm^2^.

The comparison of projected areas corresponding to *A. terreus* fully evolved pellets from the coculture and monoculture clearly indicated that *A. terreus* mycelial objects were significantly smaller when they evolved with competing *S. rimosus* (Figure S1). In 24 h of ATSR2, the difference between *A. terreus* pellets from coculture and monoculture amounted to an order of magnitude (*A. terreus* pellets: coculture A = 1.3 × 10^6^ µm^2^, monoculture A = 1.3 × 10^7^ µm^2^, *p* < 0.001).

The ATSR2 results obtained for the tested actinomycete were altogether different. The values of the projected area of *S. rimosus* fully evolved pellets (Figure 2a) in the coculture (marked with solid blue triangles) and monoculture (marked with solid red triangles) were almost identical. In 24 h, the projected area of *S. rimosus* pellets was equal to 2.9 × 10^5^ µm^2^ in the coculture and 5.1 × 10^5^ µm^2^ in the monoculture. Therefore, after the first day of the experiment, *S. rimosus* objects were the largest and their sizes showed the most significant difference between co- and mono-culture (*p* < 0.001). Next, a downward trend was observed. The final projected area for *S. rimosus* pellets equaled 1.5 × 10^5^ µm^2^ in the coculture and 1.7 × 10^5^ µm^2^ in the monoculture. Therefore, the *S. rimosus* objects were very similar (*p* > 0.05) irrespective of the presence or absence of the competing *A. terreus* in the coculture. The only exception was recorded in ATSR5. In this experiment, the pellets of *S. rimosus* vanished after 96 h. This could have been related to their irregular shapes, as indicated by the values of morphology number and elongation (Figure 2d).

Figure 2a also shows the hyphal fragments and clumps grown in ATSR2 (hollow marks). This kind of morphological object appeared in 24 h of *S. rimosus* monoculture and the coculture, whereas in *A. terreus* monoculture their presence was observed later in 48 h. The hyphal fragments and clumps’ sizes fluctuated on the level of 10^5^ µm^2^ in all three ATSR2 cultivations (*A. terreus* monoculture, *S. rimosus* monoculture, *A. terreus* and *S. rimosus* coculture).

In ATSR2, *S. rimosus* gained the morphological advantage over *A. terreus* (Figure 2a). Therefore, in the next experiments from ATSR3 to ATSR6 (Figure 2b–e) the medium compositions were modified in order to weaken the aggressive *S. rimosus* (see Table 2). The last experiment conducted using the strategy of simultaneous co-inoculation with precultures (ATSR6) was run in the medium optimal for *A. terreus* growth and lovastatin biosynthesis (lactose as the sole carbon source, without ammonium sulphate and pH control). However, all these actions did not lead to a significant change in the final result of the cultivation. Comparing *A. terreus* pellets in the experiments from ATSR2 to ATSR6, their sizes were always significantly smaller in the cocultures than in the monocultures (Figure 2). Despite the minor differences in the size of the morphological objects caused by the medium modification, the following main trends were maintained in all described experiments from ATSR2 to ATSR6: the projected area of the *A. terreus* fully evolved pellets decreased due to *S. rimosus* presence in the coculture and *S. rimosus* fully evolved pellets projected area remained practically the same in mono- and co-cultures (Figure 2a–e). It can be concluded that in the experiments ATSR2-ATSR6, *S. rimosus* always gained the morphological advantage over *A. terreus.*

The next calculated parameter, namely morphology number (Mo) (Figure 3), is connected with shape and size of mycelial objects. An Mo value close to 1 corresponds with spherical and regular forms such as spores and pellets, yet an Mo value approaching 0 represents elongated hyphae. In the experiments from ATSR2 to ATSR6, the differences between the values of morphology number for *A. terreus* and *S. rimosus* were not as clear as in the case of the projected area.

Nevertheless, based on the results it can be concluded that the change of the cultivation medium caused the decrease in *A. terreus* morphology number in the monocultures (Figure 3a,e). The highest value of this parameter, equal to 0.77, was obtained in 72 h of ATSR2 for fully evolved *A. terreus* pellets in the monoculture, while the corresponding morphology number from ATSR6 was lower and equal to 0.53 (*p* *<* 0.001). In contrast to ATSR2, the highest morphology number in the ATSR6 experiment for fully evolved *A. terreus* pellets in the monoculture was only 0.61 (at t = 48 h). The statistically significant morphological changes of fully evolved *A. terreus* pellets in the monocultures were also confirmed by *p* value calculated for the highest morphology number from the first and the last experiment (72 h ATSR2 Mo = 0.77 and 48 h ATSR6 Mo = 0.61, *p* *<* 0.001), which were conducted with the use of different cultivation media. The change of the cultivation medium composition exerted the similar effect for *A. terreus* in the coculture. Morphology number for *A. terreus* fully evolved pellets in the coculture in 72 h of ATSR2 was 0.58, and in 72 h of ATSR6 was equal to 0.40 (*p* *<* 0.05). More importantly, the presented values showed that morphology number for *A. terreus* fully evolved pellets in the coculture was lower in comparison to the monoculture (e.g., ATSR2 at 72 h in the coculture Mo = 0.58; ATSR2 at 72 h in the monoculture Mo = 0.77; *p* *<* 0.05).

Considering morphology number of *S. rimosus* fully evolved pellets in the experiments ATSR2 and ATSR6 in the corresponding time points (72 h), it is seen that the mono- and coculture values from the first and the last experiment were almost identical (Figure 3a,e): (1) *S. rimosus* monoculture fully evolved pellets Mo = 0.55 at 72 h of ATSR2 and at 72 h of ATSR6 Mo = 0.52 (*p* > 0.05); (2) *S. rimosus* coculture fully evolved pellets Mo = 0.55 at 72 h of ATSR2 and Mo = 0.54 at 72 h of ATSR6 (*p* > 0.05). However, after 72 h of ATSR2 and 72 h of ATSR6, the trends of the temporal changes of morphology number varied. In the ATSR2 (Figure 3a) case, monoculture parameters remained stable and in the coculture they were slightly raised. In ATSR6 (Figure 3e), morphology number decreased until the end of the experiment in both mono- and co-culture. Moreover, in ATSR3 (Figure 3b) relatively high morphology number values were observed. For example, in 72 h of ATSR3, for *S. rimosus* fully evolved pellets in the monoculture this parameter was equal to 0.73, and its corresponding value in the coculture was 0.64. It was evident that the changes of cultivation medium composition caused an ambiguous effect on *S. rimosus* pellets morphology number. Furthermore, the presence of *A. terreus* in the cultivation did not influence this value (Figure 3). For *S. rimosus* fully evolved pellets, there was no difference between morphology number in the monoculture and in the coculture.

The morphology number of hyphal fragments and clumps from experiments ATSR2 to ATSR6 was depicted by hollow marks (Figure 3). Their values were within the range 0.1–0.35, which was in accordance with the morphology number theory. As for the elongated hyphae objects without circular shape, morphology number was closer to 0.

Elongation (E), the last calculated morphological parameter, determined the shape of the analysed objects. Its higher values corresponded to more elongated objects such as hyphae. Low values of elongation being close to 1–1.5 indicated the presence of circular morphological forms, e.g., pellets. Figure 4 shows the temporal changes of elongation in the experiments from ATSR2 to ATSR6. During the experiments, the most remarkable changes of this parameter occurred in the first 24 h, and therefore this time point was chosen for more detailed considerations. The most important insights concerned the differences between mono- and co-cultures. Despite the fluctuations of its value, the elongation of *A. terreus* fully evolved pellets was distinctly higher in the cocultures in comparison to the monocultures, which is well justified by the selected data shown in Table 3.

However, despite the general trend, the rare cases when the elongation values of *A. terreus* fully evolved pellets in the coculture were close to the values from the corresponding monoculture or even higher occurred in the experiments from ATSR2 to ATSR6. Nevertheless, they were not statistically significant, which shows the example of 120 h of ATSR5 (coculture E = 1.37; monoculture E = 1.54; *p* > 0.05). Furthermore, the values presented in Figure 4 and Table 3 proved the lack of influence of the change of cultivation medium on the elongation *A. terreus* fully evolved pellets from ATSR2 to ATSR6 experiments.

Unlike *A. terreus*, the values of elongation for *S. rimosus* fully evolved pellets did not significantly differ in the mono- and co-cultures (Figure 4). For instance, in 24 h of the ATSR2 run, elongation of *S. rimosus* fully evolved pellets was equal to 1.36 in coculture and 1.29 in monoculture (*p* > 0.05). The corresponding elongation values from 24 h of ATSR6 reached 1.37 in coculture and 1.36 in monoculture (*p* > 0.05). Moreover, these values showed that the change of the cultivation medium composition had no influence on the elongation of *S. rimosus* fully evolved pellets. Its value from 24 h of ATSR2 coculture and ATSR6 monoculture were identical (E = 1.36; E = 1.36; *p* > 0.05). The only different trend was observed in ATSR5 (Figure 4d). In this experiment, the values of elongation of *S. rimosus* fully evolved pellets in 24, 48 and 72 h were significantly higher in the coculture than in the corresponding monocultures. The value of this parameter calculated for 24 h was equal to 1.41 in the coculture and 1.21 in the monoculture (*p* *<* 0.05). Next, *S. rimosus* pellets were progressively irregular. In 96 h of ATSR5 coculture their elongation reached 1.84, and within the next 24 h the pellets completely vanished.

The elongation for the hyphal fragments’ and clumps’ in the experiments from ATSR2 to ATSR6 fluctuated from 1.75 to 3.25, and as in the case of the morphology number, its values were in accordance with the theory.

### 3.2. Delayed Introduction of S. rimosus to Bioreactor Coculture

**Figure 5:**
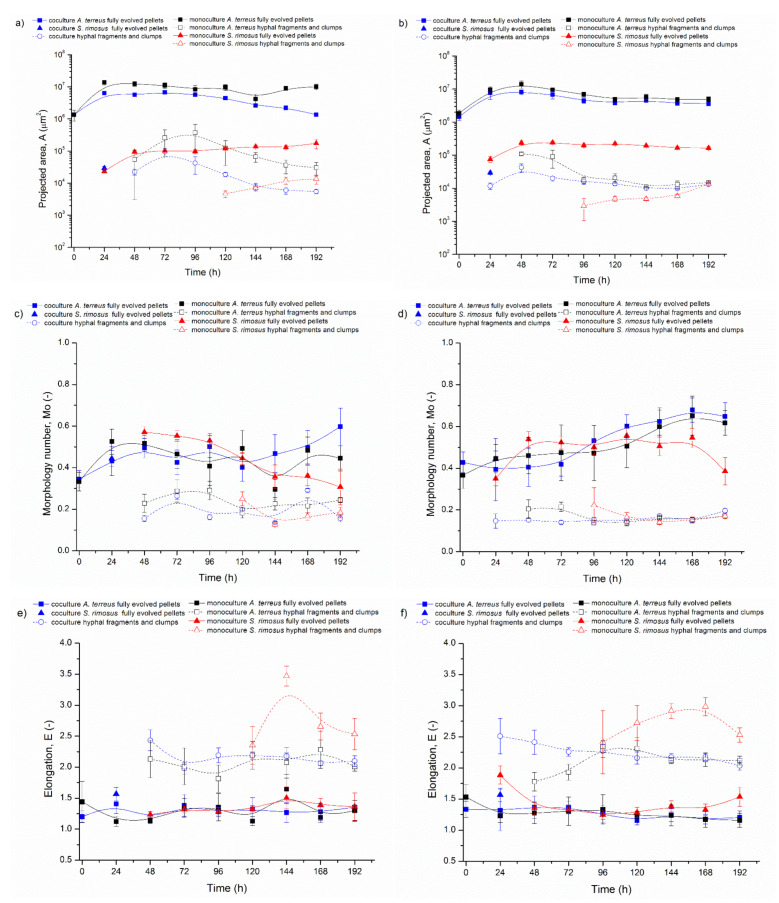
Morphological Parameters Temporal Changes in the Experiments ATSR7 and ATSR8: Projected area (A) (**a**) ATSR7 and (**b**) ATSR8, Morphology Number (Mo) (**c**) ATSR7 and (**d**) ATSR8, Elongation (E) (**e**) ATSR7 and (**f**) ATSR8.

The second stage of the study included two experiments, namely ATSR7 and ATSR8 (Figure 5). In this part, the coculture initiation method was based on the delayed introduction of *S. rimosus* to ultimately give chance of better *A. terreus* development. For this purpose, the 24 h *A. terreus* precultures were prepared and introduced to two bioreactors intended for the coculture and monoculture, respectively. After 24 h of undisturbed growth of *A. terreus*, the 24 h precultures of *S. rimosus* were introduced to the coculture bioreactor containing the fungus and to the sterile bioreactor purposed for the monoculture. ATSR7 and ATSR8 experiments differed with regard to the carbon source in the cultivation medium (lactose as the sole carbon source in ATSR7, lactose and glucose as carbon sources in ATSR8, as shown in Table 2).

Figure 5a,b present the changes of projected area of the mycelial objects in ATSR7 and ATSR8, respectively. In the moment of the introduction of *S. rimosus* preculture to the coculture bioreactor in 24 h of ATSR7, the size of *A. terreus* fully evolved pellets in the coculture bioreactor was equal to 6.3 × 10^6^ µm^2^, while in the respective monoculture bioreactor the value of 1.4 × 10^7^ µm^2^ was recorded. After next 24 h of the experiment, projected area of *A. terreus* pellets decreased to 5.6 × 10^6^ µm^2^ in the coculture bioreactor and remained at the similar level of 1.2 × 10^7^ µm^2^ in the respective monoculture bioreactor (*p* *<* 0.001). The presence of the actinomycete was the reason of this statistically significant difference.

In the ATSR8 run, the values of projected area of *A. terreus* fully evolved pellets in co- and mono-culture were even more similar than in ATSR7 in 24 h in the moment of *S. rimosus* introduction. Their exact projected area had following values: in the coculture A = 7.6 × 10^6^ µm^2^ and in the monoculture A = 9.4 × 10^6^ µm^2^ (*p* > 0.05), and their difference was not found to be statistically significant. Later on in ATSR8 coculture, 24 h after the introduction of *S. rimosus* preculture, projected area of *A. terreus* fully evolved pellets increased only to the value of 8.2 × 10^6^ µm^2^, while in the monoculture it reached 1.4 × 10^7^ µm^2^ (*p* *<* 0.01) and the difference was significant. This indicated the action of *S. rimosus*. The addition of the second carbon source (glucose and lactose used simultaneously) in ATSR8 prevented the final projected area decrease observed for *A. terreus* fully evolved pellets in ATSR7 coculture. *A. terreus* fully evolved pellets projected area at 192 h of ATSR7 coculture was equal to 1.4 × 10^6^ µm^2^, while at 192 h of ATSR8 coculture it was equal to 3.7 × 10^6^ µm^2^.

Despite the smaller size of *A. terreus* pellets in the coculture compared to the monoculture, the fungal morphological domination over *S. rimosus* in ATSR7 and ATSR8 was noticeable (Figure 5a,b, Figure S2). Although in the monocultures of *S. rimosus* the morphological changes with regard to the size of fully evolved pellets were the same as in ATSR6 (the experiment with simultaneous coculture initiation method, Figure 2e), actinomycete morphology in the cocultures was entirely dispersed. *S. rimosus* fully evolved pellets in ATSR7 and ATSR8 were only observed in 24 h, shortly after coculture initiation (solid blue triangle in Figure 5a,b). These were the ones formed in the preculture. In the next hours of ATSR7 and ATSR8, the cocultures contained only *A. terreus* pellets and hyphal fragments and clumps (hollow blue circles Figure 5a,b) from both microorganisms. For this reason, only the hyphal fragments and clumps morphological fraction was described in detail. Before ATSR7 *S. rimosus* pellets were destroyed, their projected area was equal to 3.0 × 10^4^ µm^2^ in the moment of the introduction to the coculture bioreactor. Within the next 24 h, *S. rimosus* morphology in the coculture became dispersed and the projected area of hyphal objects, not pellets, decreased to 2.2 × 10^4^ µm^2^. However, one should keep in mind that in the coculture, hyphal and clumps fraction contained both *A. terreus* and *S. rimosus*. Afterwards, in 72 h the size of hyphal and clumps fraction increased reaching maximum projected area equal to 9.4 × 10^4^ µm^2^. This value was similar and almost as high as the corresponding projected area of *S. rimosus* fully evolved pellets in ATSR7 monoculture, which was equal to 1 × 10^5^ µm^2^ (*p* > 0.05). Nevertheless, the other two morphological parameters, morphology number (Figure 5c) and elongation (Figure 5e) confirmed that the objects in 72 h of ATSR7 described as hyphae and clumps were classified correctly despite their relatively big sizes. Exact values of morphology number and elongation were 0.26 and 1.98 in the coculture and monoculture, respectively. Similar correlations between morphological parameters were observed for fraction of *A. terreus* hyphae and clumps in 72 and 96 h of ATSR7 monoculture. In terms of hyphae and clumps’ fraction distribution, the results of the ATSR8 run were the similar to the ATSR7 run (Figure 5b,d,f). In this case, only one difference was found to be significant. In 24 h of ATSR8 coculture, all three fractions (*A. terreus* pellets, *S. rimosus* pellets, hyphae and clumps) were present while in ATSR7 only the pellets of both organisms were observed.

In summary, comparing the experiments ATSR2-ATSR6 with ATSR7 and ATSR8, it can be concluded that the application of the alternative coculture initiation method caused remarkable changes in the evolution of *S. rimosus* and *A. terreus* morphology (Figures S1 and S2). The modification of cultivation medium composition also had an influence on the conducted experiments, however this effect was noticeably weaker.

### 3.3. Bioreactor Cocultures Initiated by Simultaneous Introduction of A. terreus and S. rimosus Spores

**Figure 6:**
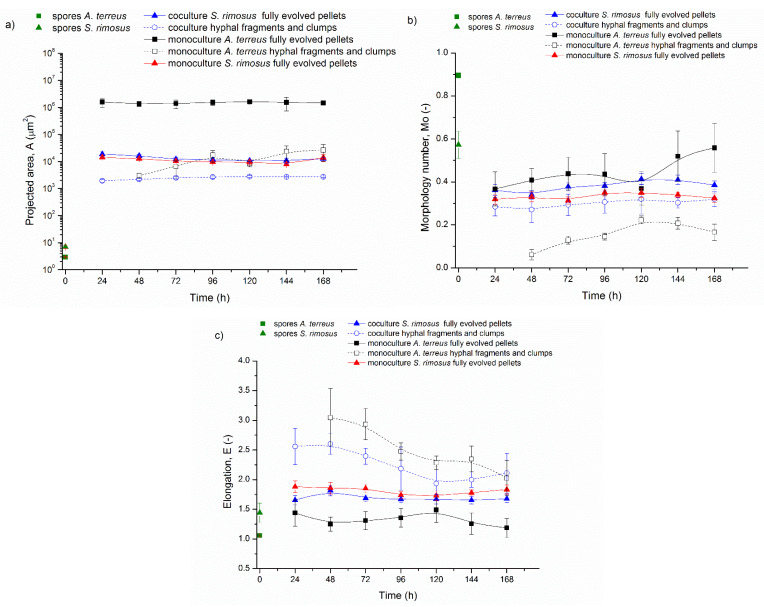
Morphological Parameters Temporal Changes in the Experiment ATSR9: (**a**) Projected Area (A), (**b**) Morphology Number (Mo), (**c**) Elongation (E).

The last tested coculture initiation method was implemented in the ATSR9 experiment (Figure 6 and Figure S3). In this experiment, *A. terreus* and *S. rimosus* spores were washed directly into the inoculation bottles and introduced simultaneously into the bioreactors intended for the coculture and monoculture controls. Moreover, this initiation method (spores versus spores) was tested in only one medium (Table 2) due to the minor morphological effect of the culture medium composition modifications observed in the previous experiments.

**Figure 7:**
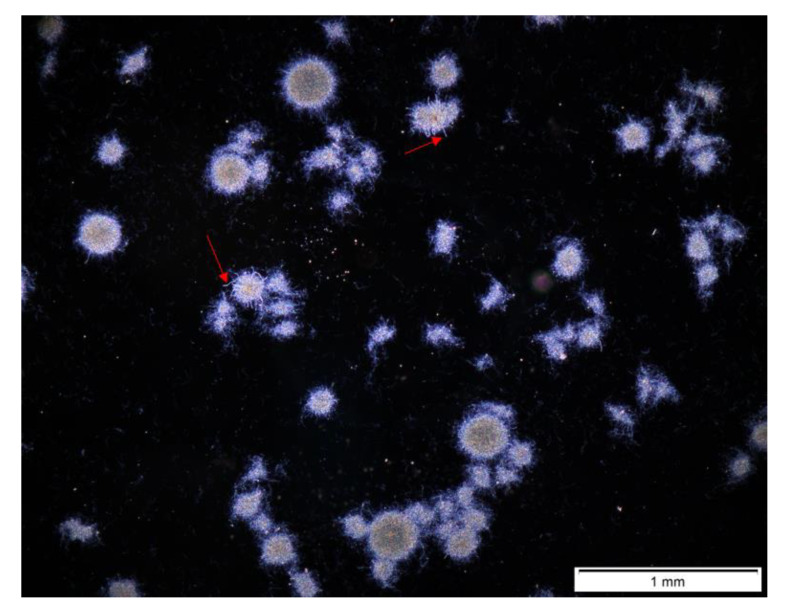
ATSR9 Coculture Mycelial Objects Snapped in 24 h of the Experiment. Red Arrows Indicate Probable *A. terreus* Hyphae Elements Integrated with *S. rimosus* Pellets.

In the ATSR9 coculture, only hyphal fragments and clumps and *S. rimosus* fully evolved pellets were present. *A. terreus* fully evolved pellets were not observed at all (Figure 6a). This indicated the morphological domination of *S. rimosus* over *A. terreus*. Furthermore, after 72 h of the experiment, *S. rimosus* pellets from the coculture had almost identical sizes to its fully evolved pellets in monoculture, which was proved by following values: The projected area of *S. rimosus* fully evolved pellets in 24 h of ATSR9 was equal 1.9 × 10^4^ µm^2^ in the coculture and 1.4 × 10^4^ µm^2^ in monoculture (*p* *<* 0.01). Therefore, the values were similar, yet the difference between them was statistically significant. Afterwards, *S. rimosus* fully evolved pellets sizes were almost equal. In 72 h of ATSR9 the corresponding projected area reached 1.2 × 10^4^ µm^2^ in the coculture and 1.0 × 10^4^ µm^2^ in the monoculture (*p* > 0.05), whereas in the end of the ATSR9 (168 h) run, projected area values amounted to 1.3 × 10^4^ µm^2^ in the coculture and 1.4 × 10^4^ µm^2^ in monoculture (*p* > 0.05). Therefore, the results obtained from the ATSR9 experiment clearly indicated *S. rimosus* having morphological domination over *A. terreus*. What is more, in rare cases the fungus was assimilated by *S. rimosus* pellets (Figure 7). It can be supposed that the thicker hyphal elements presented by red arrows in Figure 7, which were entirely integrated with *S. rimosus* pellets, might have belonged to *A. terreus*. Moreover, morphology number at 24 h of ATSR9 (Figure 6b) for fully evolved pellets in the coculture and those in the *S. rimosus* monoculture were similar and equal to 0.36 and 0.37, respectively, with *p* > 0.05. Last but not least, the values of elongation presented in Figure 6c confirmed that these objects belonged either to the fraction of *S. rimosus* fully evolved pellets or hyphae and clumps fraction. In ATSR9, they ranged from 1.86 (*S. rimosus* monoculture at 48 h) to 1.19 (*A. terreus* monoculture at 168 h) for pellets and from 1.93 (coculture at 120 h) to 3.05 (*A. terreus* monoculture at 48 h) for hyphae and clumps’ fraction.

To conclude, three described coculture initiation methods induced characteristic morphological effects. The spores-versus-spores coculture initiation method caused the morphological domination of *S. rimosus* manifested by the inhibition of the development of *A. terreus* pellets (Figure S3c) Admittedly, the preculture-versus-preculture method of coculture initiation also supported *S. rimosus*, however, it did not cause *A. terreus* pellets to vanish (Figure S1c). Therefore, *S. rimosus* was not the dominant microorganism, yet it gained the advantage over *A. terreus*. In contrast, the method of delayed introduction of *S. rimosus* to the coculture supported *A. terreus* morphological development and its domination, due to the total destruction of *S. rimosus* pellets (Figure S2c).

### 3.4. Air Flowrate and Stirring Speed Influence on Microbial Morphology in Cocultures

The application of the fully controlled bioreactors enabled measuring and tracking of the changes in air flowrate and stirring speed in each conducted experiment. Their changes were required to hold the desired level of dissolved oxygen in the cultivation broth. Both aeration and stirring were largely responsible for the mechanical forces acting on the cultivated microorganisms. This study provided interesting information regarding these issues, although it was not the main aim to investigate the effect of the mechanical forces on the development of the *A. terreus* and *S. rimosus* cocultures.

**Figure 8:**
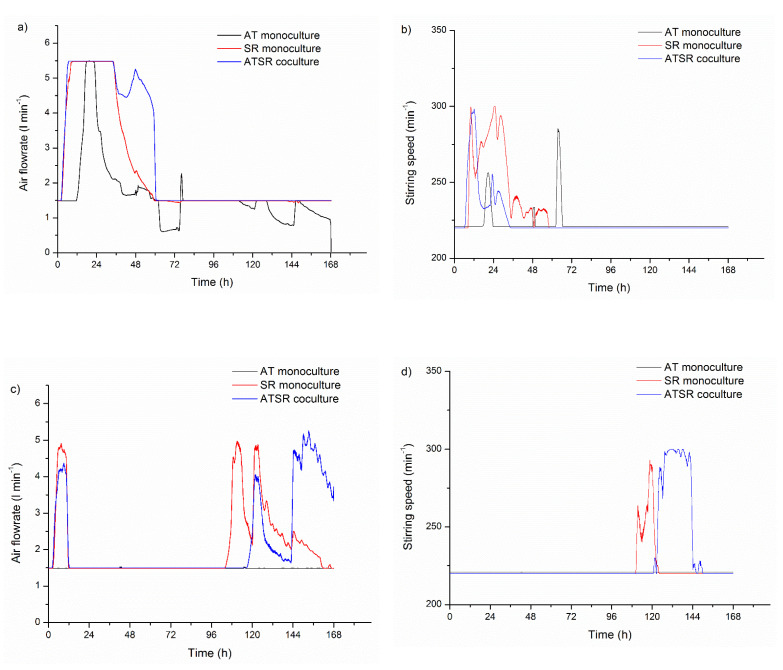
Temporal Changes of Air Flowrate (**a**) and Stirring Speed (**b**) in the Experiment ATSR2 and Temporal Changes of Air Flowrate (**c**) and Stirring Speed (**d**) in the Experiment ATSR6.

Figure 8 shows the temporal changes of air flowrate and stirring speed in ATSR2 (Figure 8a,b) and ATSR6 (Figure 8c,d). In ATSR2, the most intensive aeration and agitation occurred in the first period of the experiment. The maximum air flowrate 5.5 l_air_ min^−1^ was achieved almost immediately after the inoculation of *S. rimosus* monoculture and coculture (Figure 8a), and was required to hold the desired level of dissolved oxygen at 20%. It also reflected the relatively fast growth of *S. rimosus* due to glucose consumption (Boruta et al., 2021). The decrease in the air flowrate began after 36 h of these cultivations. In ATSR2 *A. terreus* monoculture, the maximum air flowrate was observed in the shorter period, between 17 and 22 h of the process. Stirring speed (Figure 8b) increased to its maximum value of 300 min^−1^ within 10 h of *S. rimosus* monoculture and 11 h of coculture. In the ATSR2 *A. terreus* monoculture, the first significant increase in stirring speed appeared in 20 h (255 min^−1^) and its maximum value equal to 284 min^−1^ was obtained in 63 h. After 77 h of ATSR2, both air flowrate and stirring speed remained at the lowest permissible levels in *A. terreus* monoculture. Regarding the experiment ATSR6, the changes of air flowrate and stirring speed were different in comparison to ATSR2. Apart from the temporary air flowrate increase in the first day of ATSR6 coculture and *S. rimosus* monoculture, the most intensive aeration (Figure 8c) and agitation (Figure 8d) occurred after 96 h of ATSR6. This was connected with the delayed consumption of lactose, being the sole carbon source in this experiment, by *S. rimosus* (Boruta et al., 2021). In this experiment, the maximum air flowrate was observed at 111 h of *S. rimosus* monoculture (5.0 l_air_ min^−1^) and at 153 h of the coculture (5.0 l_air_ min^−1^). In *A. terreus* monoculture, air flowrate remained at 1.5 l_air_ min^−1^ without any changes. Stirring-speed temporal changes in ATSR6 showed a similar course (Figure 8d). In *A. terreus* monoculture, its value was at the lowest set level equal to 220 min^−1^ throughout the experiment. The highest stirring speed appeared in 118 h of *S. rimosus* monoculture (292 min^−1^) and between 130 and 138 h of the coculture (300 min^−1^). Connecting the aforementioned aeration and agitation data (Figure 8) with the size of mycelial objects defined by the projected area (Figure 2a,e), it can be found that the increase in air flowrate and stirring speed in the conducted experiments led to the formation of the dispersed hyphae and clump forms. In ATSR2 *S. rimosus* monoculture and in the coculture, in which aeration (Figure 8a) and agitation (Figure 8b) were the most intensive at the beginning of the experiment, the hyphae and clumps’ fraction appeared relatively quickly, within 24 h of the experiment. On the other hand, in ATSR6 *S. rimosus* monoculture and the coculture, in which the initial increase in the air flowrate (Figure 8c) and stirring speed (Figure 8d) were not as intensive as in ATSR2, the hyphae and clumps’ fraction was noticed only in 96 h. In ATSR2 (Figure 2a) and ATSR6 (Figure 2e) runs of *A. terreus* monocultures, dispersed morphological objects appeared in 48 h. The same time point of the hyphae and clumps’ fraction appearance indicated that the mechanical forces caused by applied air flowrate and stirring speed (Figure 8) were too weak to introduce any drastic changes in *A. terreus* morphology. Moreover, the courses of air flowrate changes in *S. rimosus* monoculture and coculture were analogous in both ATSR2 and ATSR6. These similarities between the *S. rimosus* monoculture and the coculture confirmed the advantage of *S. rimosus* over *A. terreus*, which was proven previously within our quantitative morphological analysis.

## 4. Discussion

The experiments presented in this paper were described in terms of microbial morphology. Furthermore, the production of secondary metabolites and bioprocess kinetics was also tested in detail by Boruta et al. [30], which allows for a wider discussion of the relationship between morphology and metabolism of the cocultivated filamentous microorganisms. In the experiments from ATSR2 to ATSR6, where the cocultures were initiated by the parallel introduction of precultures to the bioreactors (preculture versus preculture, Figure 1, Figure 2, Figure 3 and Figure 4), *S. rimosus* gained the morphological advantage over *A. terreus*, however its domination was not as strong as in the ATSR9 experiment. This was demonstrated by a significant decrease in the *A. terreus* pellets’ projected area in cocultures (e.g., 24 h of ATSR2 *A. terreus* pellets’ projected area: coculture A = 1.3 × 10^6^ µm^2^, monoculture A = 1.3 × 10^7^ µm^2^, *p* < 0.001) while the sizes of the *S. rimosus* pellets remained the same in the mono- and co-cultures (e. g. 168 h of ATSR2 *S. rimosus* pellets’ projected area: coculture 1.5 × 10^5^ µm^2^, monoculture A = 1.7 × 10^5^, *p* > 0.05). The total ion chromatograms presented by Boruta et al. [30] showed that metabolic profiles of the broth composition of the cocultures and corresponding *S. rimosus* monocultures in ATSR2-ATSR6 were very similar. Particular attention should be paid to two *S. rimosus* metabolites, oxytetracycline and rimocidin, which show antibacterial and antifungal activity, respectively. Their levels in mono- and co-cultures were comparable. Therefore, the actinomycete was the metabolically dominant microorganism, and despite the differences in the monoculture and coculture metabolic profiles, *S. rimosus* metabolism was not repressed by *A. terreus* presence in the bioreactor. Furthermore, several new chemical compounds, not detected in the corresponding monocultures, were identified (e.g., the modified forms of rimocidin, milbemycin A_3_ derivative with two additional oxygen atoms).

In the ATSR9 experiment, in which the coculture was initiated by the parallel introduction of *A. terreus* ans *S. rimosus* spores, the actinomycete was the dominant microorganism both morphologically (Figure 6) and metabolically [30]. In this experiment, *S. rimosus* morphological domination was complete since it caused vanishing of the *A. terreus* pellets. Moreover, *S. rimosus* metabolism was not repressed by *A. terreus* at all. As with the previously described experiments from ATSR2 to ATSR6, oxytetracycline and rimocidin levels in the mono- and co-cultures were similar [30].

Only the delay in *S. rimosus* introduction to ATSR7 and ATSR8 cocultures caused the change of the prevailing microorganism from the actinomycete to the fungus, which dominated morphologically and metabolically. *A. terreus* morphological domination was total due to the absence of *S. rimosus* pellets in the coculture. According to Boruta et al. [30] *A. terreus* develops more slowly and is “less aggressive” than *S. rimosus*. Therefore, the time provided for *A. terreus* by delayed *S. rimosus* introduction in the coculture allowed for the development of fungal biomass and strengthening of *A. terreus* defense against the rival actinomycete. As a result, in ATSR7 and ATSR8 cocultures, *S. rimosus* pellets introduced to the bioreactor were destroyed during the first day of the experiment (Figure 5). Even though *A. terreus* pellets in the cocultures were smaller than pellets observed in corresponding monocultures (e.g., ATSR8 24 h *A. terreus* pellets projected area: coculture A = 7.6 × 10^6^ µm^2^, monoculture A = 9.4 × 10^6^ µm^2^, *p* > 0.05) the fungus was the dominant microorganism in ATSR7 and ATSR8, which was also confirmed by the total ion chromatograms presented in the work of Boruta et al. [30]. Oxytetracycline, *S. rimosus* metabolite, was not detected in ATSR7 and ATSR8 cocultures, while it was present in corresponding *S. rimosus* monocultures. Therefore, in ATSR7 and ATSR8 cocultures, *A. terreus* was stimulated to produce new compounds, e.g., 1-(2’,6’-dimethylphenyl)-2-n-propyl-1,2-dihydropyridazine-3,6-dione; aspereusin D; 7-deoxy-7,14-didehydro-12-acetoxy-sydonic acid or nigerapyrone [30].

Other methods of selecting the “winner” microorganism were also developed. They are based on the comparing analysis of the process parameters connected with microorganisms’ oxygen consumption. In the present paper, air flowrate and stirring speed profiles were compared (Figure 8). The similarity of these data obtained from *A. terreus* and *S. rimosus* coculture with one of the two corresponding monocultures indicated on the dominant microorganism. Dissolved oxygen profiles were used in the same way by Boruta et al. [30].

Returning to other studies on microbial morphology in the cocultures, the literature review conducted in the Introduction section revealed a complete lack of research on this issue. Despite the usefulness of quantitative morphological analysis in experiments on filamentous microorganisms [39,40,41], there are no articles that implemented the calculation of morphological parameters for cocultures of filamentous microorganisms run in stirred tank bioreactors.

This research revealed that the development of cocultures can be, with certain approximation, considered as a new morphological engineering technique, separate to the ones (such as microparticle-enhanced cultivation or salt-enhanced cultivation) described previously in the literature [42]. This method satisfied the definition of morphological engineering techniques, as the cocultivated microorganism was the factor changing the morphology. Here, the only difference was that this change of morphology was not directed to maximize the formation of selected metabolite, unlike the aforementioned previously described techniques, but influenced the whole metabolic repertoire of one of the cocultured microorganims.

Summing up, the coculturing of the filamentous microorganisms altered the biosynthetic capabilities not only by means of chemical interactions but also through morphological changes.

## 5. Conclusions

Upon the results of this study, the following conclusions can be drawn. In the *A. terreus* and *S. rimosus* cocultures, the microbial morphology depends to the highest extent on the applied coculture initiation strategy. In contrast, the modifications of cultivation medium composition (with regard to carbon and nitrogen source) do not contribute to the significant morphological changes of the studied filamentous microorganisms.

Parallel initiation strategy of the coculture leads to the dominance of *S. rimosus* over *A. terreus*. *A. terreus* gains the morphological domination only when *S. rimosus* is introduced with delay to the developed culture of *A. terreus*. This dependence is due to the slower morphological development and “less aggressive” metabolism of *A. terreus*.

Morphology and secondary metabolism of cocultured *A. terreus* and *S. rimosus* are strictly related. The microorganism which gains the morphological advantage or domination in coculture also becomes metabolically dominant.

## Figures and Tables

**Table 1:** Reported Results of *Aspergillus* and *Streptomyces* Cocultures.

	Reported Results of the Cocultures	Cocultured Strains	Cultivation Method	References
1	Mixture of *A. proliferans* and *S. olivaceoviridis* spores in the coculture provoked germination and growth of the cultivated microorganisms in medium without carbon source	*Aspergillus proliferans* *Streptomyces olivaceoviridis*	Flasks without shaking	Siemieniewicz and Schrempf [13]
2	Lecanoric acid, F-9775A, F-9775B	*Aspergillus nidulans* *Streptomyces hygroscopicus*	Shaking flasks	Schroeckh et al. [3]
3	Fumiformamide, N,N′-((1Z,3Z)-1,4-bis(4-methoxyphenyl)buta-1,3-diene-2,3-diyl)diformamide	*Aspergillus fumigatus* *Streptomyces peucetius*	Shaking flasks	Zuck et al. [31]
4	Fumicycline A (activation of a silent polyketide synthase gene cluster)	*Aspergillus fumigatus* *Streptomyces rapamycinicus*	Petri dishes	König et al. [16]
5	Ergosterol, brevianamide F, spirotryprostatin A, 6-methoxy spirotryprostatin B, fumitremorgin C and its 12,13-dihydroxy derivative, fumitremorgin B, verruculogen, 11-*O*-methylpseurotin A and its new isomer 11-*O*-methylpseurotin A_2_	*Aspergillus fumigatus MBC-F1-10 Streptomyces bullii*	Shaking flasks	Rateb et al. [32]
6	Cyclic dipeptide cyclo(Phe-Phe), 2-hydroxyphenylacetic acid, (E)-2-(3-hydroxyprop-1-en-1-yl)-phenol, (2E,4E)-3-(2-carboxy-1-hydroxyethyl)-2,4-hexadienedioxic acid	*Aspergillus niger* *Streptomyces coelicolor*	Shaking flasks	Wu et al. [17]
7	Rosellichalasin, aspochalasin E, aspochalasin P, aspochalasin H, aspochalasin M, 19,20-dihydro-aspochalasin D	*Aspergillus flavipes* *Streptomyces sp.*	Shaking flasks	Yu et al. [15]
8	Poliketide GTRI-02	*Aspergillus niger N402* *Streptomyces coelicolor A3(2)*	Shaking flasks	Wu et al. [33]
9	Luteoride D, luteoride derivative, pseurotin G, pseurotin derivative, terezine D, 11-O-methylpseurotin A, lasso peptide chaxapeptin, the titre of chaxapeptin was doubled, pentalenic acid, brevianamide X.	*Aspergillus fumigatus MR2012 Streptomyces leeuwenhoekii strain C34* and *strain C58*	Shaking flasks	Wakefield et al. [34]
10	Penicisteroid C	*Aspergillus niger* *Streptomyces piomogenus*	Shaking flasks	Abdel-Razek et al. [35]
11	The aflatoxin B1 concentration reduction by *S. roseolus*, *A. flavius* morphological changes	*Aspergillus flavus* *Streptomyces roseolus*	Petri dishes	Caceres et al. [14]
12	Heronapyrrole B	*Aspergillus sp. CMB-AsM0423* *Streptomyces sp.* *CMB-M0423*	Microbioreactor system	Khalil et al. [36]
13	Fumigermin	*Aspergillus fumigatus: Af293, A1163, ATCC 46645, IBT 16806,* *ΔfgnA, tetOn-fgnA,* *Aspergillus nidulas: RMS011, fgnA_ATCC, fgnA_Af293, fgnA_A1163_repaired, fgn_cluster,* *Streptomyces iranensis HM35, Streptomyces lividans PM02, Streptomyces coelicolor A(3)2, Streptomyces rapamycinicus ATCC 29253*	Shaking flasks	Stroe et al. [18]

**Table 2:** Cultivation Medium Compositions.

	**ATSR2**	**ATSR3**	**ATSR4**	**ATSR5**	**ATSR6**	**ATSR7**	**ATSR8**	**ATSR9**
	g·L^−1^							
lactose	0	20	20	20	20	20	20	20
glucose	20	20	20	20	0	0	20	0
yeast extract	5	2	4	4	4	4	4	4
(NH_4_)_2_SO_4_	2	2	0	0	0	0	0	0
KH_2_PO_4_	1.51	1.51	1.51	1.51	1.51	1.51	1.51	1.51
pH	7—value controlled during experiment	7—value controlled during experiment	7—value controlled during experiment	initial value 6.5; without pH control	initial value 6.5; without pH control	initial value 6.5; without pH control	initial value 6.5; without pH control	initial value 6.5; without pH control
Other components added to each culture	Salt components: 1.51 g·L^−1^ KH_2_PO_4_, 0.51 g·L^−1^ MgSO_4_·7H_2_O, 0.4 g·L^−1^ NaCl, 1 mg·L^−1^ ZnSO_4_·7 H_2_O, 2 mg·L^−1^ Fe(NO_3_)_3_·9H_2_O 0.04 mg·L^−1^ biotin A 1 mL l^−1^ of the trace elements solution was added to each culture. It contained: 100 mg·L^−1^ H_3_BO_3_, 50 mg·L^−1^ MnSO_4_, 250 mg·L^−1^ CuSO_4_·5H_2_O, 50 mg·L^−1^ Na_2_MoO_4_·2H_2_O.							

**Table 3:** Comparison of Elongation Values of *A. terreus* Fully Evolved Pellets in Cocultures and Monocultures.

24 h	E of Fully Evolved *A. terreus* Pellets in Coculture	E of Fully Evolved *A. terreus* Pellets in Monoculture	*p* Value
ATSR2	1.56	1.08	*p<* 0.001
ATSR3	1.61	1.19	*p<* 0.01
ATSR4	1.68	1.16	*p* = 0.05
ATSR5	1.50	1.14	*p<* 0.05
ATSR6	1.40	1.16	*p<* 0.001

## Data Availability

The data used to support the findings of this study are available from the corresponding author upon request.

## Supplementary Materials

The following are available online at https://www.mdpi.com/article/10.3390/biom11111740/s1, Figure S1: Microscopic image of the morphological objects snapped in 24 h of ATSR2. Figure S2: Microscopic image of the morphological objects snapped in 24 h of ATSR8. Figure S3: Microscopic image of the morphological objects snapped in 24 h of ATSR9.
