# Supplementary material for: Quantitative Morphological Analysis of Filamentous Microorganisms in Cocultures and Monocultures: Aspergillus terreus and Streptomyces rimosus Warfare in Bioreactors
Source: Biomolecules. 2021 Nov 22;11(11):1740. doi: 10.3390/biom11111740 (PMC8615777; doi:10.3390/biom11111740)
Supplement: Supplementary file 1 [file biomolecules-11-01740-s001.zip › biomolecules-1464884-supplementary.pdf]

a)

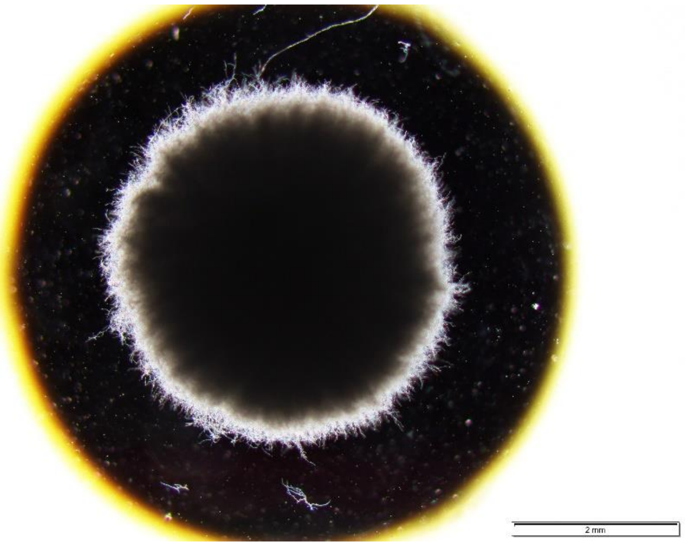

b)

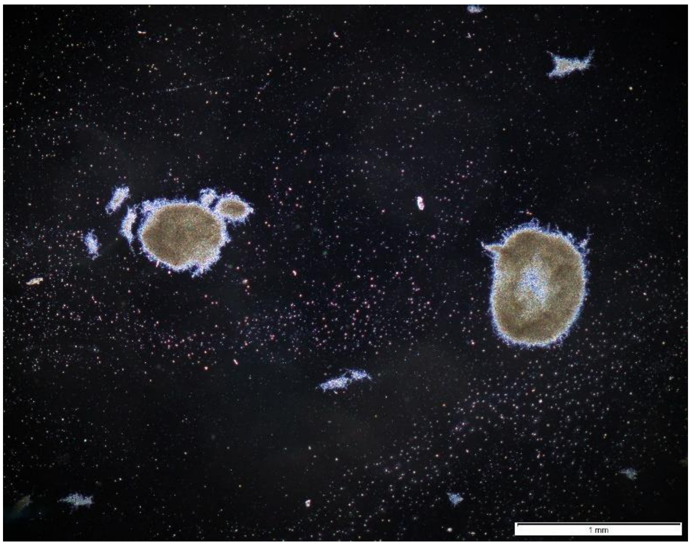

c)

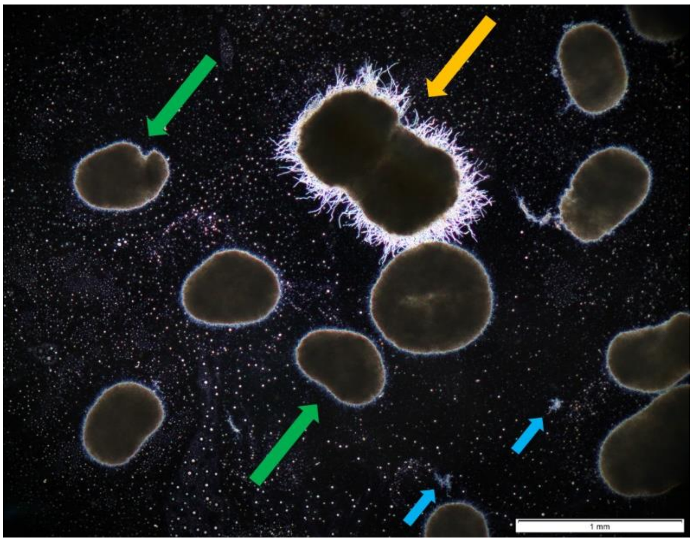

Supplementary Figure S1. Microscopic image of the morphological objects snapped in 24 h of ATSR2: (a) *A. terreus* monoculture, (b) *S. rimosus* monoculture, (c) *A. terreus* and *S. rimosus* co-culture. The co-culture morphological objects were marked as follows: *A. terreus* fully evolved pellet - yellow arrow, *S. rimosus* fully evolved pellets - green arrows, hyphae or clumps of both species - blue arrows.

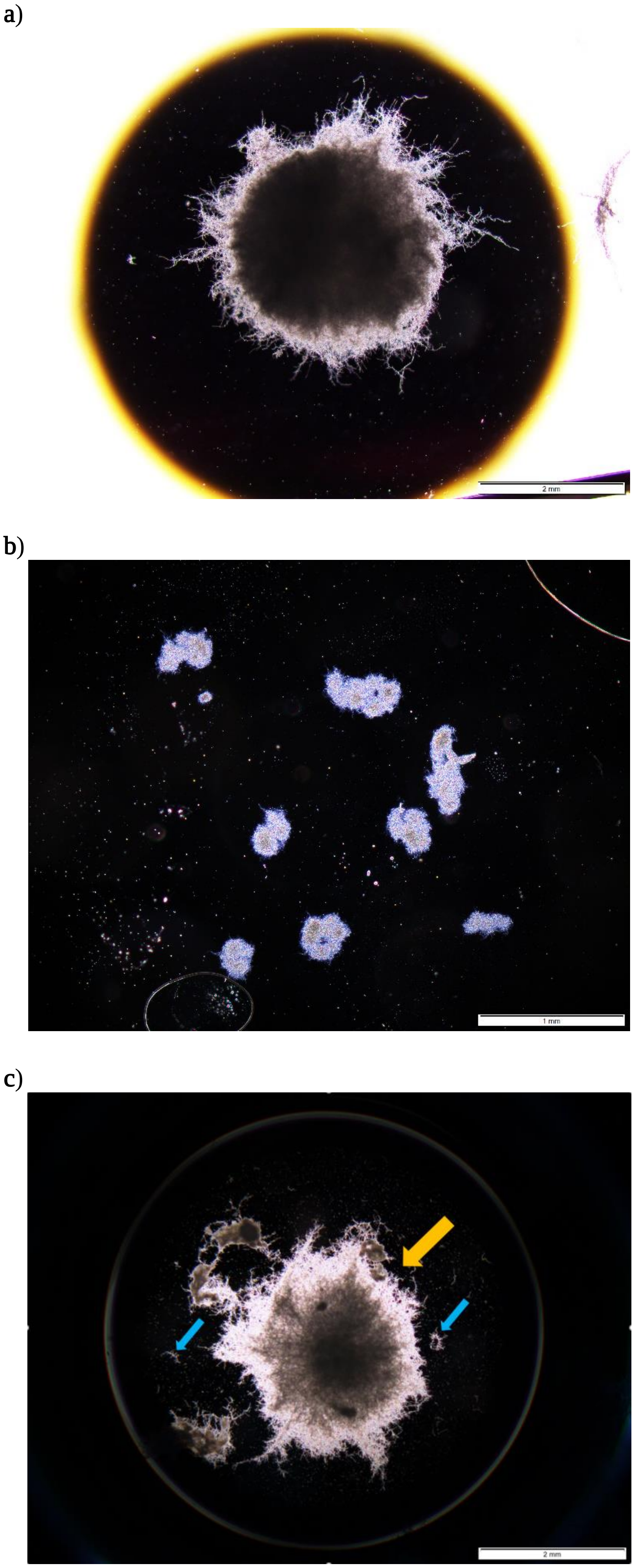

Supplementary Figure S2. Microscopic image of the morphological objects snapped in 24 h of ATSR8: (a) *A. terreus* monoculture, (b) *S. rimosus* monoculture, (c) *A. terreus* and *S. rimosus* co-culture in which *A. terreus* pellets are visible and hardly any *S. rimosus* objects can be found (*A. terreus* fully evolved pellet - yellow arrow, hyphae or clumps of both species - blue arrows).

a)

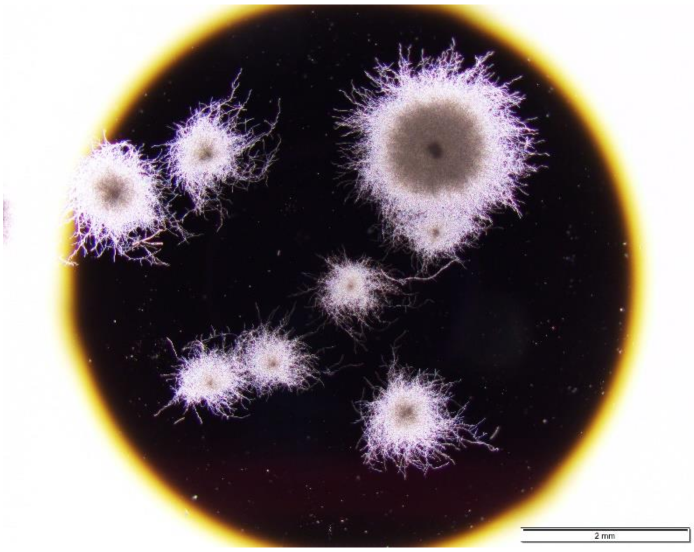

b)

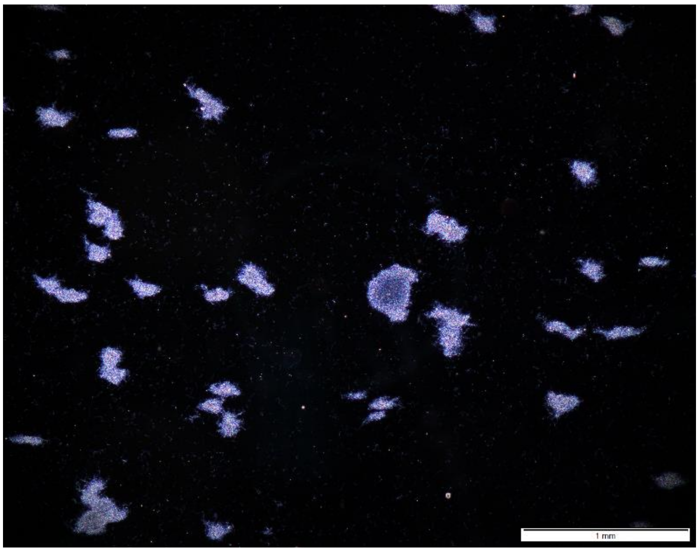

c)

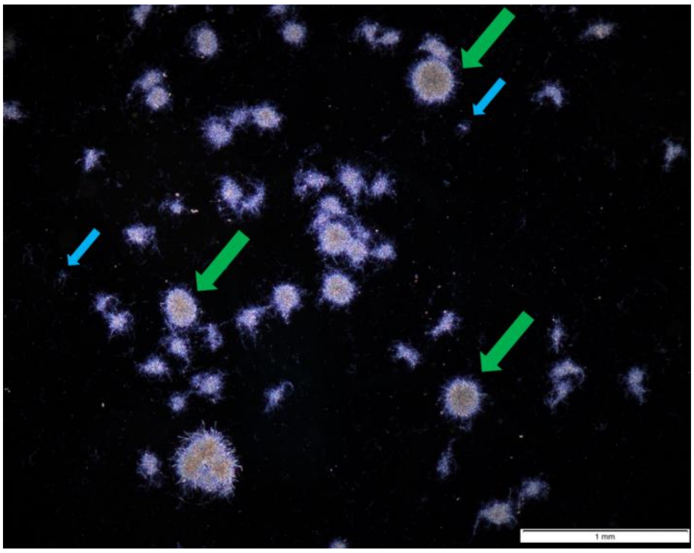

Supplementary Figure S3. Microscopic image of the morphological objects snapped in 24 h of ATSR9: (a) *A. terreus* monoculture, (b) *S. rimosus* monoculture, (c) *A. terreus* and *S. rimosus* co-culture, in which *S. rimosus* pellets are visible and hardly any *A. terreus* objects can be found (*S. rimosus* fully evolved pellets - green arrows, hyphae or clumps of both species - blue arrows).
